# Novel Technique for Placement of Immediate Implant in Mandibular Region: A Case Series

**DOI:** 10.31729/jnma.8113

**Published:** 2023-04-30

**Authors:** Farwad Silwal, Sanjeeta Katwal, Shovana Gyawali

**Affiliations:** 1 Sanjivani Dental Care Center, Biratnagar, Nepal; 2 Department of Dental Surgery, Nepal Army Hospital, Itahari, Nepal

**Keywords:** *case reports*, *extraction*, *nobel technique*, *osseointegration*

## Abstract

Immediate implant placement is an approach to immediately insert a dental implant into newly created extraction socket following tooth extraction. Since osseointegration is one of the major factors for implant success, placing an immediate implant in between mesial and distal roots acts as natural surgical template and bone formation around the implant from extraction socket acts or gives better osseointegration. We reported 4 cases, in which nobel technique was used. It was used in mandibular first and second molars where we do immediate implants in case of a tooth beyond repair and in case of leftover roots. Here in the case of only root involvement, we have drilled and prepared osteotomy in between the mesial and distal root while in the case of the whole tooth, we have to section the crown first, then drill. Thus, better osseointegration with good amount of soft tissue formation above the implant was achieved.

## INTRODUCTION

Placement of implants is very common around the globe. The immediate implant is the placement of an implant immediately after extraction of the deceased tooth which is commonly done nowadays. Thus, we have reported four cases presenting chief complaints like fractured teeth and abscesses, thus after Cone Beam Computed Tomography (CBCT), the immediate implant was planned. For cases involving root only we have to drill and prepare osteotomy in between the mesial and distal root while in the case of the whole tooth, we have to section the crown first and then drill. Thus, better osseointegration was achieved. However, a wider application of this technique for longer following up periods is required for further conclusive recommendations.

## CASE 1

**Figure 1:**
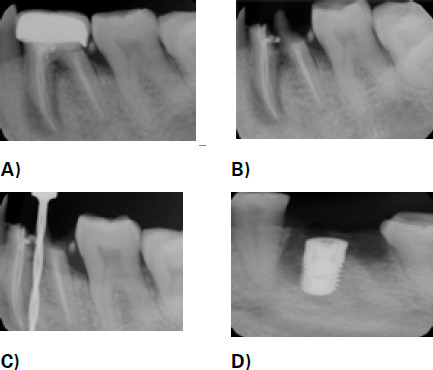
Radiographic image showing A) artificial crown with periapical radiolucency extending till furcation area and fracture of distal root at the level of the crest, B) sectioning of crown horizontally and separation in between mesial and distal roots, C) After osteotomy, checking for center point in between mesial and distal roots, D) Implant placement in the desired area after removing roots.

A 59 years old female patient, came to the Dental Care Center with abscess in relation to 36 (mandibular left first molar). Previously 5-6 years back, the root canal was treated and the crown was placed. On examination, tenderness on percussion was present and sinus tract formation was seen. Intraoral periapical radiograph, 36 revealed a fracture from the furcation area to the distal root, an abscess from the furcation area to the mesial root (Figure 1).

The patient was advised for an immediate implant and Cone Beam Computed Tomography (CBCT) of the mandible to measure the vertical bone density along with vertical and horizontal bone length. Thus, based on a CBCT report, implant size was determined. As per the evaluation, 4/10 mm of Bredent implant was planned to be placed. Furthermore, sectioning of the coronal portion and osteotomy in between the roots. While performing the procedure at first: An inferior alveolar nerve block was given using lidocaine hydrochloride 2% with Adrenaline 1:80,000. The coronal portion of the tooth was sectioned. Drilling started in between the mesial and distal roots with normal burs using an airotor and then drilling with burs from the implant surgical kit. After the final drill, 4/10 mm of Bredent implant and cover screw were placed. The torque achieved was 30 nm. On follow-up, infiltration was given using the same local anesthesia. The minimum gingiva was removed and the gingival former (healing abutment) was placed.

Thus, the Implant stability quotient was measured with a penguin. The patient recalled after 1 week for an impression. On the third follow-up, the impression was made with elastomeric impression material (light buddy and putty) for porcelain fused to a metal crown. On the final visit, the crown was cemented in the abutment and screwed on the implant.

## CASE 2

**Figure 2:**
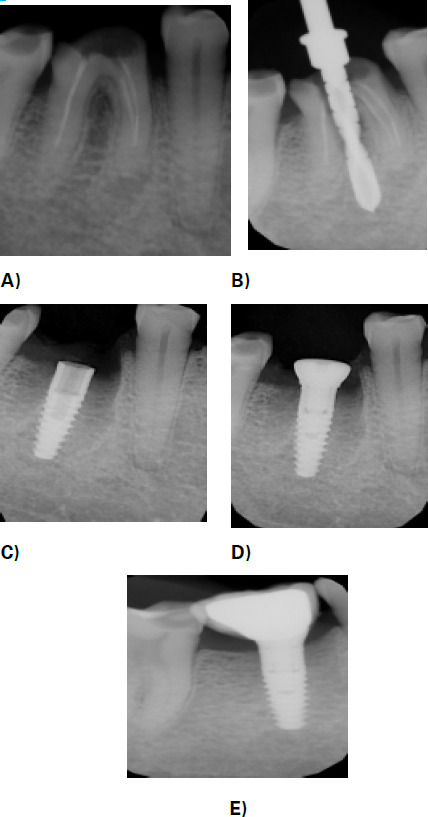
Radiographic image showing A) loss of coronal structure with linear radiopacity short of apex on both the roots along with file separation on mesial root, B) After osteotomy, checking for center point in between mesial and distal roots,C) Placement of implant on the drilled area after removing roots D) Gingival former placement on implant, E) Placement of crown on implant after 3 months.

A 51 years old male patient presented to the dental care center with chief complaints of a fractured tooth while chewing food in the lower right back teeth region. The tooth was RCT (root canal treatment) treated a few years back. On examination, the coronal portion of the lower first molar was fractured. In the intraoral periapical radiograph, periapical pathology was not see (Figure 2).

The patient was advised immediate implant in relation to 46 (right mandibular first molar) and CBCT of the mandible. The treatment plan as per the CBCT evaluation decided to place 3.5/12 mm of bredent implant. Thus, the procedure was performed by giving the Inferior alveolar nerve block using lidocaine hydrochloride 2% with Adrenaline 1:80,000. Drilling started after sectioning the mesial and distal roots till the furcation area and the center point was achieved. Furthermore, the drilling was started using the pilot drill, secondly, a twisted drill was used. Now a parallel pin was placed in the drilled area and IOPAR was taken to check if it is in the center or not. Then a 3.5 mm final drill was used. After the final drill, a 3.5/12 mm implant was placed. Here the gingival former was placed for better tissue closure and then a suture was placed. The patient was recalled after 1 week for suture removal. After 3 months the patient was revisited for a prosthesis. The implant stability quotient was measured by penguin. It was found to be more than 70. Then the impression was taken as similar to other implant cases. The Crown was cemented after a few days.

## CASE 3

**Figure 3:**
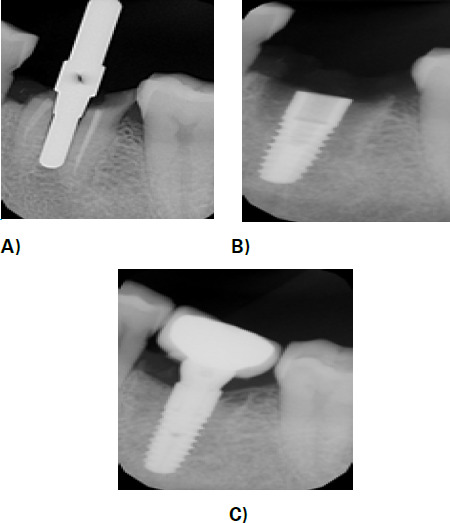
Radiographic image showing A) medial position achieved after initial osteotomy exactly between mesial and distal roots, B) implant placed after final drill in exact center point after roots removal, C) crown placed after 3 months.

A 58 years old female patient, presented to the dental clinic with chief complaints of a fractured tooth in the lower right back region. The tooth was RCT treated 8 years back. On examination, the whole coronal portion was fractured. The intraoral periapical radiograph showed the remaining radicular portion with no periapical changes (Figure 3). The patient was advised of immediate implant placement in relation to 46 (right mandibular first molar). The treatment plan as per CBCT evaluation was to place an implant of size 3.5/12 mm. The procedure was performed by giving the Inferior alveolar nerve block using lidocaine hydrochloride 2% with Adrenaline 1:80,000. Drilling started after sectioning the mesial and distal roots till the furcation area and the center point was achieved. Furthermore, the drilling was started using the pilot drill, secondly, the twisted drill was used. Now a parallel pin was placed in the drilled area and IOPAR was taken to check if it is in the center or not. Then a 3.5mm final drill was used. After the final drill, a 3.5/10 implant was placed. Here, the gingival former was placed for better tissue closure and then sutured. The patient was recalled after 1 week for suture removal. After 3 months the patient was revisited for a prosthesis. The implant stability quotient was measured by the penguin after removing the gingival former. It was found to be 73. Then the impression was taken as similar to other implant cases. The Crown was cemented after 7 days.

## CASE 4

A female aged 60 years presented to the Sanjivani dental care center with chief complaints of a fractured tooth in the lower right back teeth region.

**Figure 4:**
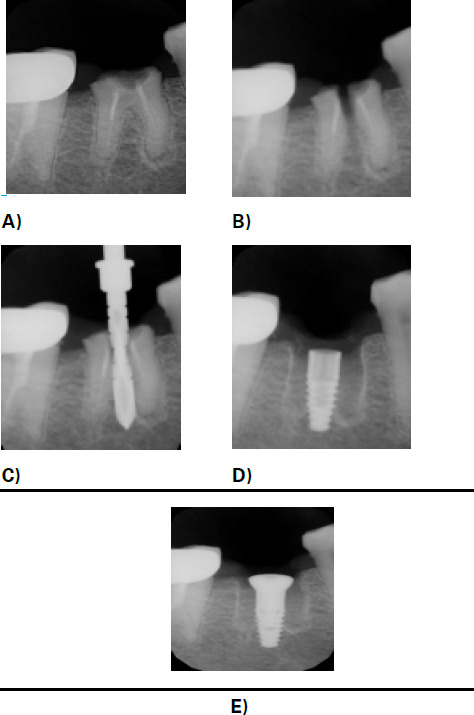
Radiographic image showing A) remaining roots with under obturated canals with no coronal portion, B) separation of mesial and distal roots till furcation, C) achieving center point after initial osteotomy in between mesial and distal roots, D) placement of implant in exact point after roots removal, E) radiograph showing gingival former placement.

The tooth was RCT treated 10-12 years back, and the patient was advised for a crown a few years back. On Examination, the coronal portion of the lower right first molar was fractured. For investigation, IOPAR was taken (Figure 4). It showed under obturated canal with the absence of a coronal portion. The patient was advised immediate implant in relation to 46 (right mandibular first molar) and CBCT of the mandible. The treatment plan as per the CBCT evaluation decided to place 3.5/10 mm of bredent implant. An inferior alveolar nerve block was given using lidocaine hydrochloride 2% with Adrenaline 1:80,000. Drilling started after sectioning the mesial and distal roots till the furcation area and the center point was achieved. Furthermore, the drilling was started using the pilot drill, secondly, a twisted drill was used. Now a parallel pin was placed in the drilled area and IOPAR was taken to check if it is in the center or not. Then a 3.5 mm final drill was used. After the final drill, a 3.5/10 implant was placed. Here the gingival former was placed for better tissue closure and then a suture was placed. The patient was recalled after 1 week for suture removal. After 3 months the patient was revisited for a prosthesis. The implant stability quotient was measured by penguin. It was found to be more than 70. Then the impression was taken as similar to other implant cases. The Crown was cemented after a few days.

## DISCUSSION

For the patient, losing a tooth in the aesthetic region is frequently a terrible event. The most affordable and long-lasting option for replacing missing teeth with a high average life expectancy is a dental implant.^3^ Dental implant at present is designed to replicate the natural tooth root and crown of the natural tooth. This procedure preserves the gingival mucosa and bone with no damage to adjacent teeth.^4^ Immediate implant placement refers to placing a dental implant in an extraction socket at the moment of extraction or explantation, while delayed implant placement refers to placing an implant in a location that is edentulous after healing and new bone development have taken place.^1^ In this study, we have reported 4 case studies, in which the novel technique of immediate dental implant was placed. About three of the patients presented with chief complaints of a fractured tooth in the lower right back teeth region while one of the patients had abscess in relation to 36 (mandibular left first molar). In another study, the case had a similar presentation of fractured upper front teeth due to trauma.^5^ All patients had history of root canal therapy previously. In our case we have planned for the immediate implant after CBCT scan. While in another study, pre-surgical radiographic evaluation was carried out with IOPA, panoramic radiograph and CT Dentascan.^5^ In our case, only the involvement root, we have drilled and prepared osteotomy in between the mesial and distal root while in the case of the whole tooth, we have sectioned the crown first and then drilled. The procedure was performed by giving the Inferior alveolar nerve block using lidocaine hydrochloride 2% with Adrenaline 1:80,000. Drilling started after sectioning the mesial and distal roots till the furcation area and the center point was achieved. In another study, after local anesthesia, the fractured tooth was traumatically removed. The extraction socket was thoroughly debrided and after sequential drilling the implant was placed in the socket with the insertion torque of 45 Ncm.

Implant first thread was placed 1.5 mm apical to the crestal bone of the socket and adequate primary stability was obtained.^5^ Furthermore, in our cases, the drilling was started using the pilot drill, secondly a twisted drill was used. Now a parallel pin was placed in the drilled area and IOPAR was taken to check if it is in the center or not. After achieving center point roots were removed atraumatically. Then a 3.5mm final drill was used. After the final drill, a 3.5/10 implant was placed in the prepared interradicular area. Here, the gingival former was placed for better tissue closure and then sutured. The success rate for immediate implant placement in the fresh extraction socket is high in recent research.^6^

The roots act as a natural surgical guide to place the implant in a central position. Surgical guide or template is not required that minimizes the cost of the procedure. It helps to maintain the parallel axial axis of the implant to that of adjacent teeth. It is only applicable in immediate implant placement. It cannot be done in severe infection cases. It's difficult to perform in convergent roots. In case of close proximity to an inferior alveolar nerve canal, it cannot be performed. The combination of immediate implant placement with the engagement of the interseptal/ inter radicular bone showed preliminary favorable outcomes. However, a wider application of this technique for longer following up periods is required for further conclusive recommendations.
